# Silver-Doped Reduced Graphene Oxide/PANI-DBSA-PLA Composite 3D-Printed Supercapacitors

**DOI:** 10.3390/nano14201681

**Published:** 2024-10-20

**Authors:** Claudia Cirillo, Mariagrazia Iuliano, Davide Scarpa, Pierpaolo Iovane, Carmela Borriello, Sabrina Portofino, Sergio Galvagno, Maria Sarno

**Affiliations:** 1 Department of Physics “E.R. Caianiello”, University of Salerno, Via Giovanni Paolo II, 132-84084 Fisciano, Italy; maiuliano@unisa.it (M.I.); dscarpa@unisa.it (D.S.); msarno@unisa.it (M.S.); 2 NANO_MATES Research Centre, University of Salerno, Via Giovanni Paolo II, 132-84084 Fisciano, Italy; 3 Nanomaterials and Devices Laboratory (SSPT-PROMAS-NANO), ENEA, Italian National Agency for New Technologies, Energy and Sustainable Economic Development, Piazzale E. Fermi 1, 80055 Portici, Italy; pierpaolo.iovane@enea.it (P.I.); carmela.borriello@enea.it (C.B.); sabrina.portofino@enea.it (S.P.); sergio.galvagno@enea.it (S.G.)

**Keywords:** silver-doped reduced graphene oxide, polyaniline–DBSA composite, 3D printing supercapacitors, fused deposition modeling, electrochemical performance, additive manufacturing

## Abstract

This study presents a novel approach to the development of high-performance supercapacitors through 3D printing technology. We synthesized a composite material consisting of silver-doped reduced graphene oxide (rGO) and dodecylbenzenesulfonic acid (DBSA)-doped polyaniline (PANI), which was further blended with polylactic acid (PLA) for additive manufacturing. The composite was extruded into filaments and printed into circular disc electrodes using fused deposition modeling (FDM). These electrodes were assembled into symmetric supercapacitor devices with a solid-state electrolyte. Electrochemical characterization, including cyclic voltammetry (CV) and galvanostatic charge–discharge (GCD) tests, demonstrated considerable mass-specific capacitance values of 136.2 F/g and 133 F/g at 20 mV/s and 1 A/g, respectively. The devices showed excellent stability, retaining 91% of their initial capacitance after 5000 cycles. The incorporation of silver nanoparticles enhanced the conductivity of rGO, while PANI-DBSA improved electrochemical stability and performance. This study highlights the potential of combining advanced materials with 3D printing to optimize energy storage devices, offering a significant advancement over traditional manufacturing methods.

## 1. Introduction

In recent years, additive manufacturing (AM)—also known as rapid prototyping (RP) or solid freeform fabrication (SFF)—has gained increasing attention and importance across various industrial sectors. Introduced by Charles Hull in 1986, AM has revolutionized the creation of three-dimensional objects, allowing for precise and customized manufacturing from computer-generated 3D models [1]. By utilizing a layer-by-layer deposition or polymerization techniques, AM enables complex and detailed geometries that are often inaccessible with traditional methods [2]. Unlike subtractive manufacturing, which involves removing material from an initial block and inevitably generating wastes, AM significantly reduces material waste, minimizes environmental impact, and lowers production costs [3]. Furthermore, AM drastically shortens production times, making it particularly advantageous for rapid prototyping and small-scale production [4].

In the context of the growing global demand for advanced and sustainable energy solutions, energy storage has become a critical area of focus. Supercapacitors have emerged as a key technology due to their high power density, rapid charge and discharge capabilities, and long cycle life [5]. Traditional methods for producing supercapacitors, which involve slurry preparation, coating, and drying, are energy- and time-intensive and use toxic solvents [6]. AM technologies offer an innovative solution, allowing for complex and customized electrochemical components in a single, efficient step, reducing production time and costs [7].

Among the materials studied to enhance supercapacitor performance, carbon-based materials, and in particular graphene-based ones, have shown great potential. For example, Feng et al. [8] developed a hybrid material by covalently coupling a Schiff base 2D porous polymer with graphene oxide (GO) sheets. This material, benefiting from the conductivity of graphene, achieved specific capacitance values of up to 424 F g^−1^ at 0.1 A g^−1^. Furthermore, reduced graphene oxide (rGO), obtained from the reduction of graphene oxide (GO), combines high electrical conductivity with a large surface area and improved stability, making it an ideal candidate for supercapacitor electrodes [9,10]. These properties enhance electrical permittivity and charge carrier accumulation, improving overall performance. However, the mechanical fragility of rGO presents a significant challenge in creating durable and robust 3D structures [11]. To address these challenges and optimize rGO properties, doping with metallic nanoparticles has proven to be an effective strategy [12]. Silver (Ag) is known for its excellent electrical conductivity, and it is a cost-effective alternative to noble metals like gold (Au) and ruthenium (Ru) [13]. Recent studies have demonstrated that silver nanoparticles and reduced graphene oxide exhibit enhanced electrochemical sensing properties compared to traditional materials [14]. The high density of free electrons in silver improves the electrical properties of rGO, facilitating charge storage and conductivity.

Additionally, combining graphene with conductive polymers such as polyaniline (PANI) has shown promising benefits for supercapacitor performance. PANI, with its high electrical conductivity and environmental stability, complements rGO’s properties and further enhances electrochemical performance [15]. For instance, Song et al. [16] developed self-wrinkled PANI-based composite hydrogel with a core–sheath structure to be adopted as an all-in-one supercapacitor, reaching high specific capacitances of 504 mF cm^−2^ and 210 F g^−1^ at 0.5 A g^−1^, a high energy density of 70 µW h cm^−2^, and outstanding cycling stability of 5000 charge–discharge processes. Ahirrao et al. [17] developed an innovative flexible supercapacitor using nanostructured PANI coated on a conductive carbon cloth substrate through a straightforward in situ chemical oxidative polymerization technique, achieving 691 F/g at 1 A/g, as well as 94% capacitance retention after 2000 charge–discharge cycles. However, PANI has inherent limitations, including low cycle life and poor stability during charge–discharge cycles. These issues can be mitigated by doping PANI with surfactants like dodecylbenzenesulfonic acid (DBSA) [18]. DBSA, as an ionic surfactant, improves PANI’s conductivity, stability, and solubility, addressing some of its performance drawbacks [15]. The combination of DBSA with PANI not only enhances its electrochemical performance but also contributes to the development of more robust and durable electrode materials.

In this study, we aim to develop a versatile and adaptable method for producing robust and high-performance 3D-printed supercapacitors by synthesizing a composite of silver-doped reduced graphene oxide (rGO) and DBSA-doped polyaniline (PANI). The addition of silver is intended to enhance the electrical conductivity of rGO, while DBSA-doped PANI will offer further electrochemical benefits and improved stability. This composite is then blended with polylactic acid (PLA), which is readily processable via additive manufacturing (AM). The resulting material is extruded into filaments and printed using fused deposition modeling (FDM) into circular disc electrodes, which are subsequently assembled into solid-state electrolyte-based symmetric devices. The electrochemical performance of these devices is evaluated through cyclic voltammetry (CV) and galvanostatic charge–discharge (GCD) curves. This approach not only explores innovative ways to optimize supercapacitor materials but also leverages AM technologies to advance the field of energy storage, addressing the challenges of traditional production methods and meeting future market demands [19].

## 2. Materials and Methods

### 2.1. Materials

Silver nitrate (AgNO_3_, 99%), urea (100.5%, CH_4_N_2_O), ethylene glycol (C_2_H_6_O_2_), citric acid (99.9%, C_6_H_8_O_7_), polyaniline (PANI), dodecylbenzenesulfonic acid (DBSA), polylactic acid (PLA), N-methyl-2-pyrrolidone (NMP), sulfuric acid (H_2_SO_4_), ethanol, and water were of analytical grade and obtained from Aldrich Chemical Co. (Milan, Italy).

### 2.2. Synthesis of Ag/rGO Nanocatalyst

**Figure 1:**
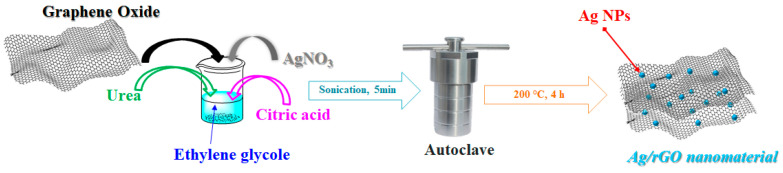
Synthesis of Ag/rGO nanomaterial by solvothermal method.

A mixture comprising 300 mg of graphene oxide (GO), 3% by weight of silver nitrate (AgNO_3_) relative to GO, 100 mg of citric acid, and 1.8 g of urea was prepared in 30 mL of ethylene glycol. The mixture underwent sonication using an ultrasound tip (Hielscher model UP400S, Teltow, Germany) at the maximum power of ultrasounds for 5 min, resulting in a black solution. This solution was transferred to a 150 mL autoclave and heated to 200 °C for 4 h, generating an internal pressure of approximately 10 bar. After completion, the solution was cooled to room temperature. The resultant black material was washed three times with ethyl alcohol and water, followed by drying at 60 °C for 12 h to obtain the final product (see Figure 1).

### 2.3. Functionalization of Ag/rGO@PANI-DBSA

The Ag/rGO nanomaterial was functionalized with the polyaniline–dodecylbenzene sulfonic acid (PANI-DBSA) complex. First, PANI in its emeraldine base form was mixed with DBSA at 140 °C for 5 min in a 1:3 weight ratio, then dried at 50 °C for 4 h. The resulting PANI-DBSA complex was dispersed in N-methyl-2-pyrrolidone to form a 30 mg/mL solution. This solution was added to 6 mL of a 16 mg/mL Ag/rGO dispersion and stirred at room temperature for 10 min, resulting in the composite Ag/rGO@PANI-DBSA.

### 2.4. Ag/rGO@PANI-DBSA-PLA Composite, 3D Printing, and Device Fabrication

Ag/rGO@PANI-DBSA nanocomposites were blended with a thermoplastic polymer known as polylactic acid (PLA), chosen for its numerous advantageous properties. PLA is considered the most widely used biodegradable thermoplastic and belongs to the aliphatic polyester category. This widespread usage stems from the fact that PLA is not only easily accessible but also biocompatible, making it highly desirable for a variety of applications. Furthermore, PLA is known for its relatively strong mechanical properties, which further enhance its appeal in manufacturing processes.

Specifically, to optimize printing performance, a specific weight ratio was determined as the most effective formulation. In this case, the composites were mixed with PLA (granules) at a precise weight ratio of 1:2 (composites to PLA). This ratio was carefully selected after thorough experimentation to ensure that the resulting mixture would have the best processability in 3D printing applications, particularly in the extrusion-based FDM process. Once the composites and PLA were thoroughly mixed, the resulting mixture was heated to a temperature of 200 °C. This temperature was chosen as it falls within the optimal processing range for PLA, ensuring the material melts evenly without degradation. During this heating phase, the mixture was continuously stirred to ensure proper homogenization, resulting in a uniform and consistent compound. After the heating and mixing process, the compound was allowed to cool to room temperature under controlled conditions to prevent any defects or irregularities in the final material.

The next phase of the procedure involved feeding the cooled, PLA-loaded composite filament into a MiniCTW twin-screw extruder, manufactured by ThermoScientific (Milan, Italy). The extrusion process was carried out at a constant temperature of 200 °C, while the screws operated at a controlled speed of 30 revolutions per minute (rpm).

The parameters were carefully optimized to produce a smooth and uniform filament with a precise diameter of 1.75 mm, which is the standard width for most 3D printing applications. The diameter refers to the measurement across the circular cross-section of the filament, ensuring consistent thickness throughout its length. Once the filament reached the desired diameter, it was coiled for storage and made ready for use in the next stage of the 3D printing process. Specifically, the Ag/rGO@PANI-DBSA nanocomposites were mixed with PLA in a 1:2 weight ratio and extruded into filaments using a twin-screw extruder at 200 °C. The mixture is physically combined rather than chemically bonded. During the heating and mixing process, the components form a uniform and homogeneous compound due to the physical attachment of the nanocomposite to the PLA matrix. This composite is then extruded into a filament suitable for 3D printing, maintaining its structural and conductive properties for use in supercapacitors

The 3D models were designed using advanced computer-aided design (CAD) software, specifically Solidworks, which is widely recognized for its ability to generate complex geometries with high precision. The 3D models were then converted into corresponding G-code files using PrusaSlicer, a slicing software that prepares the models for printing by generating the appropriate toolpaths. These G-code files were subsequently used to instruct the 3D printer, a Prusa i3 MK3S+ model (also by Prusa), to print the objects using the FDM technique. The objects were 3D printed as circular disc-shaped electrodes, each measuring 1 cm in diameter and 2 mm in thickness. These dimensions were selected to create uniform and precise discs that would serve as the foundation for constructing supercapacitor devices.

After successfully printing the conductive composite discs, these components were used to assemble devices specifically designed to function as symmetrical supercapacitors. Each device consisted of two 3D-printed discs positioned opposite one another. A solid electrolyte was placed between the two discs to create a compact sandwich-like structure. The solid electrolyte used in these devices was prepared by mixing 6 g of polyvinyl acetate (PVA) with 10 milliliters of a 1 molar sulfuric acid (H_2_SO_4_) solution. The mixture was carefully homogenized until it formed a uniform conductive electrolyte gel without any additional treatments required to form the gel, facilitating efficient charge transfer between the discs.

### 2.5. Characterization Techniques

Scanning electron microscopy (SEM) images were captured using a TESCAN-VEGA LMH (230 V) electron microscope equipped with an energy-dispersive X-ray (EDX) probe. Transmission electron microscopy (TEM) analyses were conducted using the FEI Tecnai (200 kV) instrument. The powder X-ray diffraction (XRD) patterns were recorded with a Bruker D8 X-ray diffractometer utilizing CuKα radiation. Additionally, Fourier transform infrared (FT-IR) spectroscopy was performed using a Vertex 70 apparatus (Bruker Corporation, Milan, Italy).

Electrochemical measurements of the composites, before mixing with PLA, were conducted in a 0.5 M H_2_SO_4_ electrolyte solution. For these measurements, 8 mg of the synthesized samples were dispersed in a mixture of 160 μL of a 5 wt% Nafion solution, 900 μL of 2-propanol, and 100 μL of water to form a homogeneous ink. This mixture was sonicated for 30 min and then partially deposited dropwise onto a DRP-110 screen-printed electrode (SPE) (Metrohm, Herisau, Switzerland), which consists of a carbon working electrode, a platinum counter electrode, and a silver reference electrode. SPEs were selected due to their superior properties compared to standard carbon electrodes.

Cyclic voltammetry (CV) and galvanostatic charge and discharge (GCD) tests on the electrode were carried out using an Autolab PGSTAT302N potentiostat (Metrohm, Herisau, Switzerland) in a three-electrode configuration.

The mass-specific capacitance values (Cs, F/g) were calculated from CV and GCD curves according to the following equations [20], respectively:

(1) $$C_{s}(CV)=\frac{\int_{vb}^{va}i\cdot dV}{m\cdot v\cdot ∆V}$$

(2) $$C_{s}(GCD)=\frac{i\cdot ∆t}{m\cdot ∆V}$$

where Δ*V* is the voltage range, *v* is the scan rate, *i* is the current (discharge current in the GCD), Δ*t* is the discharging time, and *m* is the active mass of the single electrode.

Furthermore, the energy density (*E*, Wh/kg) and the power density (*P*, W/kg) of the device have been evaluated, in a two-electrode configuration, according to the following equations:

(3) $$E=\frac{1000}{3600\cdot 2}Cs(GCD)\cdot \;{∆V}^{2}$$

(4) $$P=3600\frac{E}{∆t}$$

where *Cs* is the mass capacitance calculated in (2), Δ*t* is the discharging time and Δ*V* is the potential window.

## 3. Results and Discussion

### 3.1. XRD Characterization

**Figure 2:**
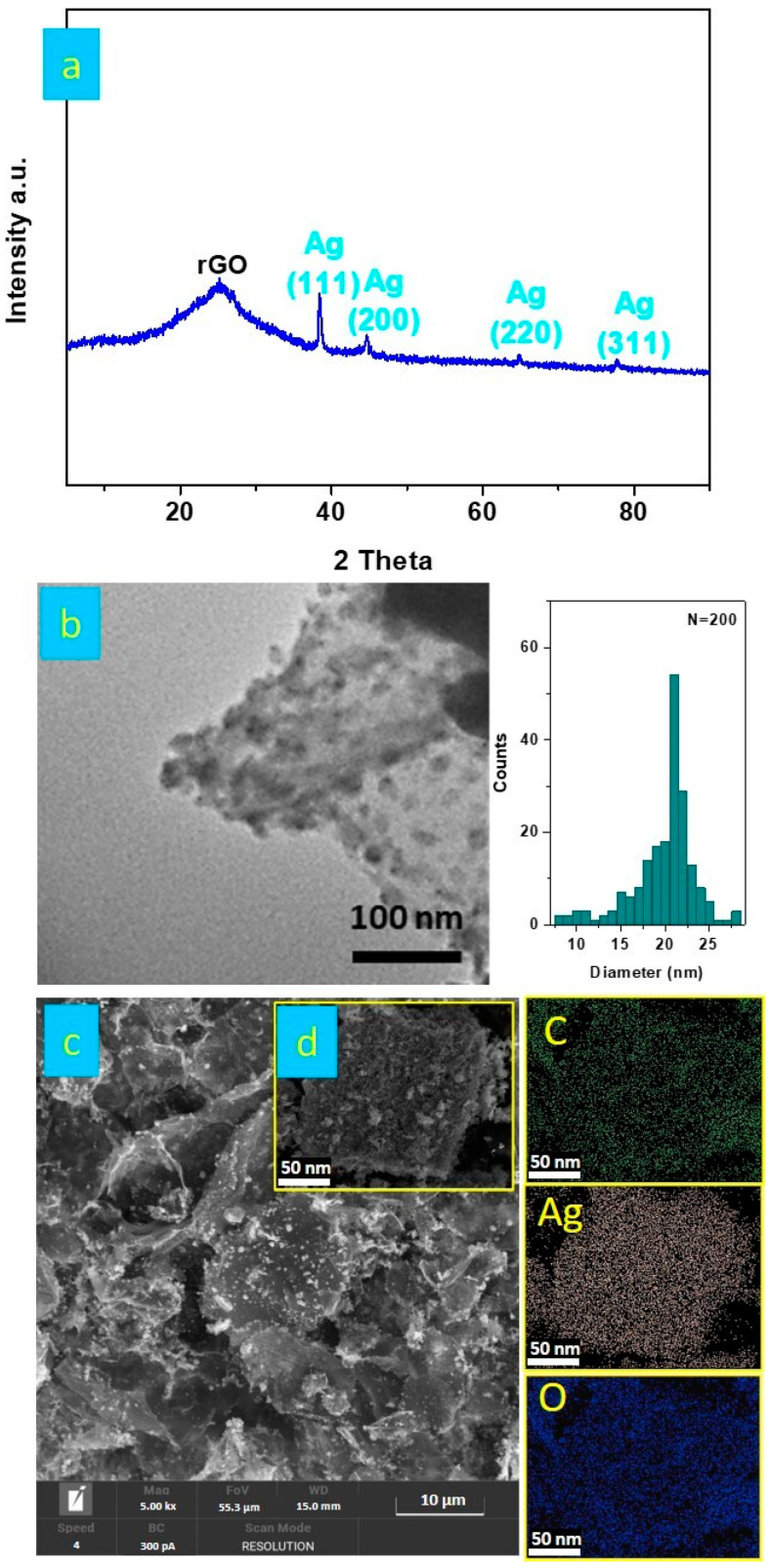
XRD spectrum of Ag/rGO nanomaterial (**a**); transmission electron microscopy (TEM) image of Ag/rGO nanomaterial with size distribution histogram (**b**); scanning electron microscopy (SEM) image of Ag/rGO (**c**); and energy-dispersive spectroscopy (EDX) elemental mapping of C, O, and Ag (**d**).

The structure of the Ag/rGO nanocomposite was analyzed using XRD, as shown in Figure 2a. The XRD pattern of the Ag/rGO nanocomposite revealed a slightly broader diffraction peak at approximately 2θ = 25°, corresponding to the (002) plane with an interlayer spacing of 0.236 nm, is associated with the presence of graphene in the hybrid material [21,22]. Additionally, peaks at 2θ = 38.2°, 44.5°, 64.5°, and 77.8° were observed, corresponding to the (111), (200), (220), and (311) planes of face-centered cubic (fcc) Ag nanomaterials with interplanar spacing (d-spacing) value of the Ag nanoparticles was 1.6 Å. No impurity was detected. The estimated particles size of the Ag/rGO nanocomposite was determined to be 21 nm, calculated from the full width at half maximum of the Ag (111) plane peak. Therefore, the XRD results of the Ag/rGO nanocomposite indicate the incorporation of rGO sheets with Ag nanoparticles.

The TEM (transmission electron microscopy) image of the Ag/rGO nanocomposite, shown in Figure 2b, displays Ag nanomaterials embedded in the rGO matrix, appearing as dark spots in the TEM image. This demonstrates that the Ag nanomaterials possess irregular shapes.

The Ag/rGO nanocomposite morphology was also observed through SEM analysis (see Figure 2c). SEM images showed Ag nanoparticles aggregates with sizes ranging from 250 to 800 nm can be observed decorating the wrinkled rGO surface, confirming the uniform distribution of Ag nanoparticles over the crinkled and corrugated rGO nanosheets.

Additionally, a color mapping analysis using SEM was conducted to determine the elements’ distribution in the Ag/rGO nanocomposite (Figure 2d). The color mapping images of the Ag/rGO nanocomposite revealed the presence of carbon (green), oxygen (blue), and silver (light pink) elements, which combined to form the Ag/rGO nanocomposite. The uniform distribution of Ag over the rGO sheets was observed, confirming the formation of Ag with rGO in the prepared Ag@rGO nanocomposite.

**Figure 3:**
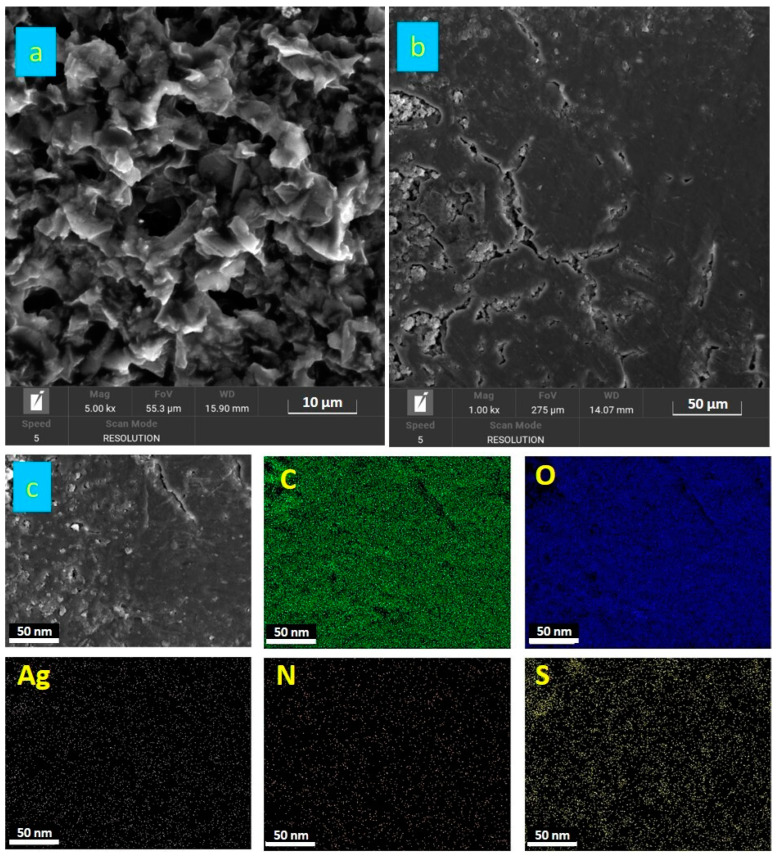
SEM images of Ag/rGO@PANI-DBSA (**a**); Ag/rGO@PANI-DBSA-PLA (**b**); and energy-dispersive spectroscopy (EDX) of Ag/rGO@PANI-DBSA-PLA nanocomposite elemental mapping of C, O, N, S, and Ag (**c**).

Figure 3a displays SEM images of Ag/rGO–PANI-DBSA nanocomposites. The low-magnification SEM micrograph shows silver deposited on rGO sheets anchored with the PANI-DBSA complex. Figure 3b explores the morphology of Ag/rGO@PANI-DBSA-PLA composites using SEM analysis, revealing rough surfaces with cracks and ridges. Figure 3c presents an SEM image (scale bar: 50 µm) and corresponding EDX maps of Ag/rGO@PANI-DBSA-PLA. These maps confirm the composite’s presence, identifying carbon (C), oxygen (O), nitrogen (N), sulfur (S), and silver (Ag) elements.

### 3.2. FT-IR Analysis

**Figure 4:**
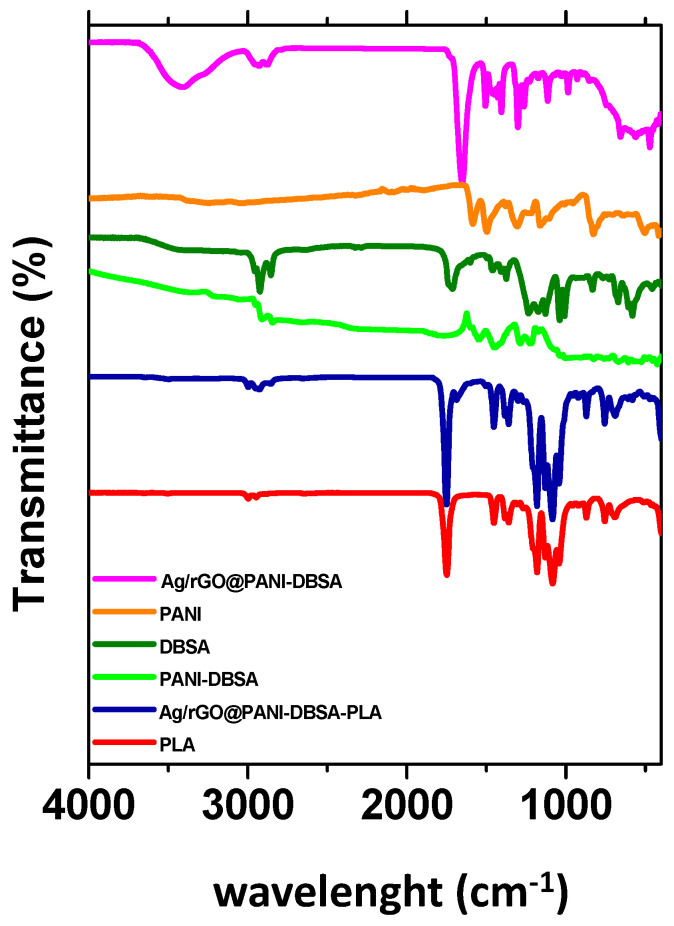
FT-IR spectra of Ag/rGO@PANI-DBSA nanomaterial, PANI, DBSA, PANI-DBSA, Ag/rGO@PANI-DBSA-PLA nanocomposite, and PLA.

The FT-IR spectra of GO and Ag/GO nanomaterials are shown in Figure S1. The broad band at 3400 cm^−1^ corresponds to the O–H stretching vibration of graphene oxide. The epoxy vibration at 1055 cm^−1^ and other oxygenated functional groups such as C=O at ~1714 cm^−1^ and C–O at ~1173 cm^−1^ are also detected. The band at ~1621 cm^−1^ indicates the C=C bonding of GO aromatic rings, which are mostly absent in Ag/GO, suggesting reduction. A small shift in Ag/GO vibration bands suggests a strong interaction between GO and Ag nanoparticles. FT-IR analysis of the conductive Ag/rGO@PANI-DBSA-PLA composite (Figure 4) confirms the presence of PLA with its CH, CH_2_, and CH₃ bonds. Intense PLA bands (red profile) mask those of Ag/rGO@PANI-DBSA. Bands at 2847–2991 cm^−1^ correspond to CH, CH_2_, and CH₃ groups in PLA, PANI-DBSA, and rGO. The 1655 cm^−1^ graphene oxide band is hidden by the 1750 cm^−1^ PLA C=O stretching band. A band at 1185 cm^−1^ indicates PLA C–O–C stretching. The PANI band shifts from 1467 cm^−1^ to 1448 cm^−1^, and the rGO C–O–C band shifts from 1216 cm^−1^ to 1213 cm^−1^, indicating interactions with PLA. The DBSA S=O band is slightly visible at 746 cm^−1^. Bands between 1000 and 500 cm^−1^ are due to –OH, C–C, C–COO, and C= vibrations of PLA. Key bands at 1750 cm^−1^ and 1599 cm^−1^ correspond to PLA C=O stretching and PANI C=N vibrations, confirming successful blending with PLA.

### 3.3. Electrochemical Characterization of Devices for Supercapacitor Applications

**Figure 5:**
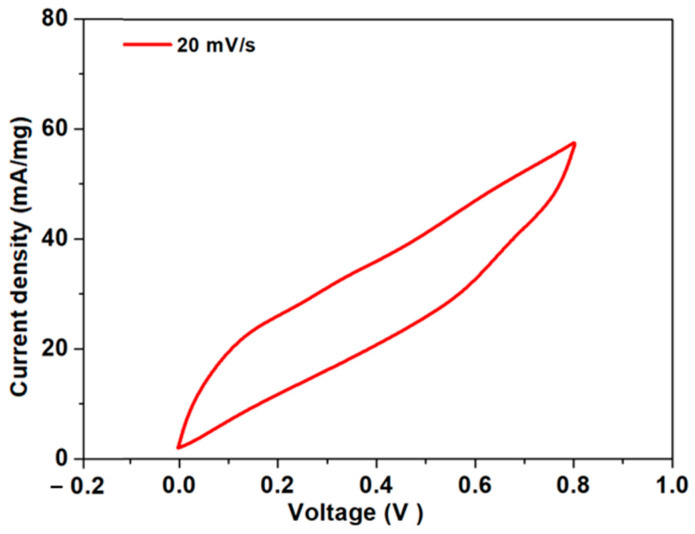
CV curve of the Ag-rGO@PANI-DBSA electrode at 20 mV/s.

Current–voltage measurements are essential for assessing the capacitance performance of the sample. For this purpose, a cyclic voltammogram was recorded within the optimal voltage range of 0.0 to 0.8 V at a scan rate of 20 mV/s. The CV profile of the Ag-rGO@PANI-DBSA electrode material, reported in Figure 5, shows a quasi-rectangular shape with no detectable redox peaks indicating a nearly ideal capacitive behavior [23,24]. From the CV curve, it can be deduced that the primary contribution to the charge storage mechanism of the synthesized material comes from the double-layer capacitance of rGO and, to a lesser extent, from the Faradaic Ag NPs and PANI [25]. By analyzing the CV polarization curve using Equation (1), the mass-specific capacitance was found to be 136.2 F/g at 20 mV/s. The Cs value of the device could be ascribed to the high-surface-area rGO flakes acting as efficient charge storage buffers. Additionally, the Ag nanoparticles on the flakes not only improve the electrical conductivity of the electrode, increasing the charging and discharging rate, [26,27] but also act as spacers between the rGO sheets, thereby preventing interlayer agglomeration and maintaining the high surface area, which would otherwise be lost due to the restacking of graphene layers [28]. Furthermore, the presence of PANI-DBSA guarantees improved wettability, enhancing the interaction between electrolyte ions and the electrode surface. After all, DBSA not only enhances the AM processability along with PLA, but also acts as a stabilizing agent by preventing the aggregation of nanoparticles and improving the electrochemical stability of PANI [29].

**Figure 6:**
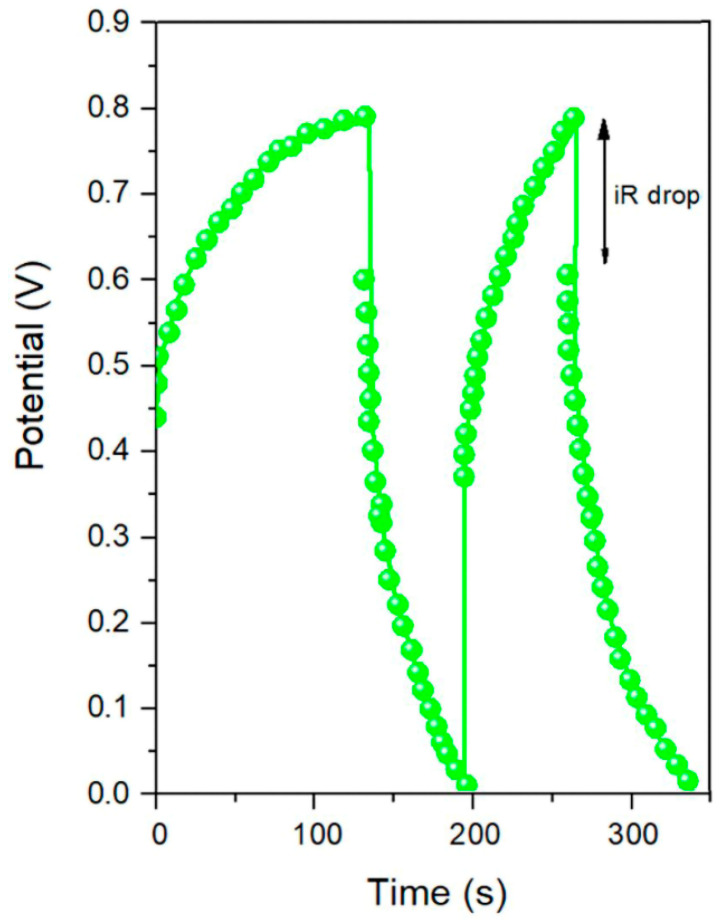
Galvanostatic charge–discharge curves of the Ag-rGO@PANI-DBSA electrode at 1 A/g.

The results of the charge–discharge test on the Ag-rGO@PANI-DBSA sample in the voltage range of 0–0.8 V at 1 A/g, reported in Figure 6, confirm the described behavior.

The Figure 6 shows slightly asymmetric triangular-shape curves, which suggest the high reversibility of the material with a small iR drop.

**Figure 7:**
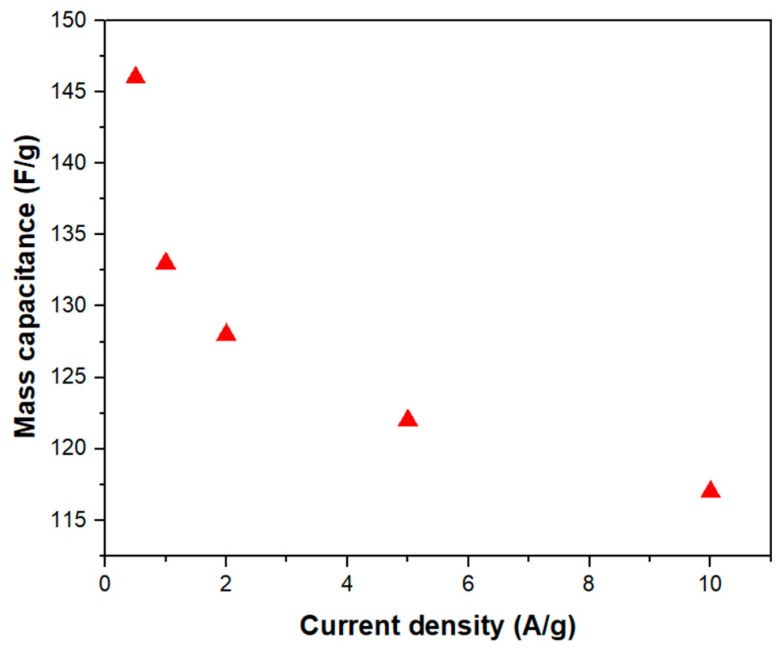
Specific capacitance vs. current density of the Ag-rGO@PANI-DBSA electrode.

The specific capacitance value, calculated from the curve according to Equation (2), is 133 F/g at 1 A/g, which can be once again ascribed to the synergy between the device components. The capacitance can be also attributed to the particle size distribution, which affects the quantity of assembled Ag NPs/reduced graphene oxide and the available active surface area for electrochemical reactions. Moreover, the presence of larger particles improves the effective ion wettability of the sample by enhancing the electrolyte accessibility between them. Figure 7 reports the mass capacitance values at different current densities, showing a decreasing trend with increasing current densities in the range of 0.5–10 A/g.

This behavior is expected because higher current densities allow shorter time intervals for electrolyte ions to diffuse into the electrode channels, thereby accessing a smaller portion of the active material’s surface area. However, capacitance remains considerable (117 F/g), even at the high discharge current density of 10 A/g, proving the efficiency of the material.

**Figure 8:**
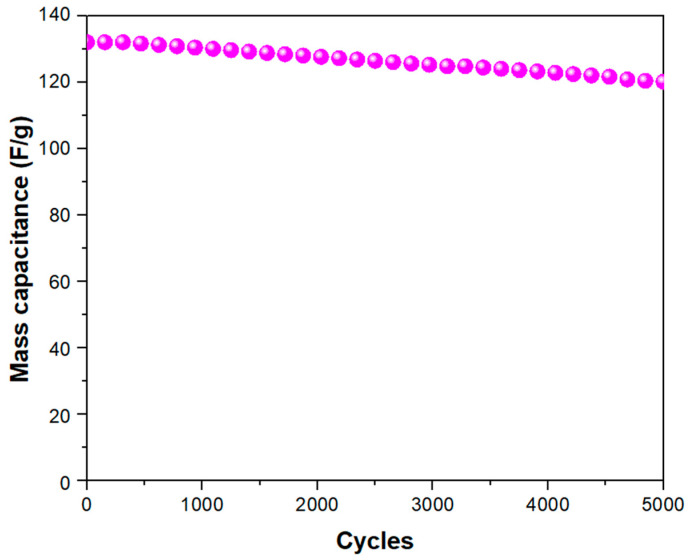
Capacitance vs. number of cycles at 1 A/g for the Ag-rGO@PANI-DBSA electrode.

Furthermore, the sample was tested after several cycles of usage, showing encouraging results in terms of durability. In particular, as reported in Figure 8, Ag-rGO@PANI-DBSA retains 91% of its initial capacitance value after 5000 cycles at 1 A/g.

The high durability can be explained by the intimate electronic connection among the components, which reduces internal resistance to charge transfer and thereby extends electrode durability, and by the presence of Ag particles in the rGO layers. Indeed, it has been reported that Ag nanoparticles can enhance mechanical strength, making the composite less prone to deterioration compared to the rGO sheets alone, which would otherwise easily break down, thus reducing its surface area and limiting electron and ion transport [28,30].

**Figure 9:**
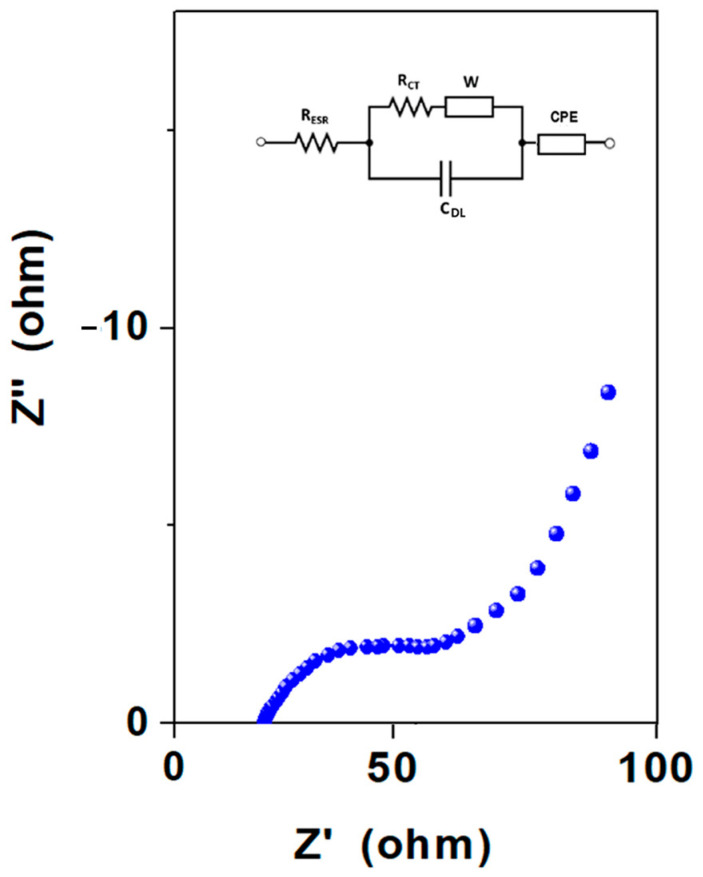
EIS results for the Ag-rGO@PANI-DBSA electrode.

Additionally, electrochemical impedance spectroscopy (EIS) was adopted to further evaluate the electrochemical performance of the synthesized material. Figure 9 displays the Nyquist plot for the Ag-rGO@PANI-DBSA sample, recorded in the frequency range 10^−1^ ÷ 10^5^ Hz at OCP, along with its corresponding electrical equivalent circuit. In the plot, an inclined line appears in the low-frequency region, followed by a quasi-semicircle in the high-frequency region. The depressed semicircle can be modeled as a charge transfer resistance at the interface between the electrode and the electrolyte (R_CT_), in parallel with the capacitance of the double layer (C_DL_). The inclined segment of the curve initially approximates 45°, representing the Warburg impedance (W), which indicates the resistance to ion diffusion from the electrolyte to the electrode surface. This segment then shifts to an almost vertical line, indicating a nearly ideal capacitive behavior, and can be represented by a constant-phase element (C_PE_). In the high-frequency region, the intercept on the Z’ axis indicates the equivalent series resistance (R_ESR_), which includes the internal resistance of the electrode, the ohmic resistance of the electrolyte, and the contact resistance between the current collector and the electrode, and is equal to about 25 Ω.

**Figure 10:**
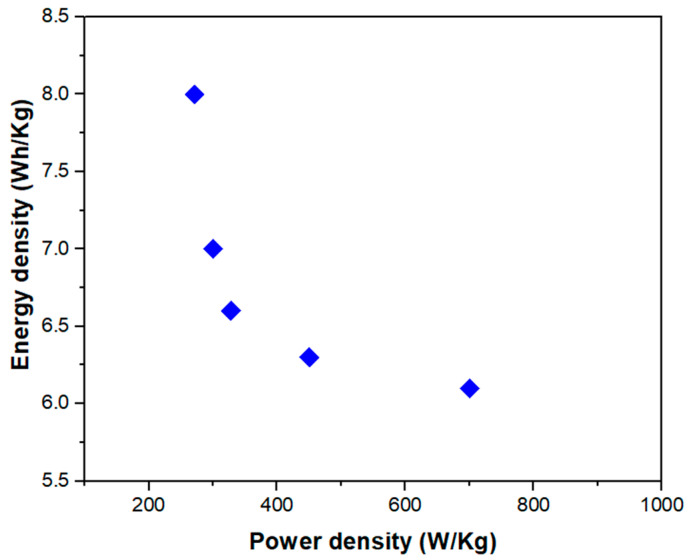
Ragone plot for the Ag-rGO@PANI-DBSA-PLA device.

Starting from the specific capacitance values, energy densities and power densities of the device were also evaluated according to (3) and (4) (see Ragone plots in Figure 10).

Values of energy and power densities were recorded, respectively, in the range of 8–6.3 Wh/kg and 282–700 W/kg, proving the good performance of the device.

The recorded performance of the material proves that the obtained results are comparable to those achieved with non-3D-printed composites of Ag-, rGO-, and PANI-based materials in the literature, even when comparing those with finer sizes, as shown in Table 1.

## 4. Conclusions

In this study, we developed an innovative and versatile method for producing high-performance 3D-printed supercapacitors using a composite of reduced graphene oxide (rGO) doped with silver and polyaniline (PANI) doped with dodecylbenzenesulfonic acid (DBSA). The combination of silver and PANI with rGO demonstrated significant improvements in electrical conductivity and electrochemical stability, enabling the creation of robust and highly efficient electrodes.

The analysis of the results revealed that silver doping not only enhanced the electrical conductivity of rGO but also improved the energy storage capacity and overall performance of the material. The presence of silver nanoparticles helped maintain a highly active surface area and prevented the agglomeration of graphene layers, while DBSA improved the solubility and stability of PANI, contributing to the greater durability of the composite.

Electrochemical measurements confirmed that the resulting devices exhibited considerable specific capacitances of 136.2 F/g at 20 mV/s and 133 F/g at 1 A/g, with energy and power densities comparable to the literature. The stability of the supercapacitors was demonstrated by maintaining 91% of their initial capacitance after 5000 charge–discharge cycles, highlighting exceptional durability.

These results suggest that integrating AM with advanced materials, such as rGO, silver, and PANI-DBSA, not only optimizes the performance of energy storage devices but also provides a significant advantage over traditional production methods. The adoption of 3D printing technologies for manufacturing supercapacitor electrodes could revolutionize the field of energy storage technology, effectively addressing the growing demand for advanced and sustainable energy solutions.

This study represents a significant step forward in combining advanced materials and additive manufacturing technologies to enhance the performance and efficiency of energy storage devices.

## Figures and Tables

**Table 1:** Supercapacitance performance of the Ag-rGO@PANI-DBSA-PLA device and some of the highest-performing non-3D-printed composites including Ag-, rGO-, and PANI-based materials in literature.

Ag-, rGO-, and/or PANI-Based Materials for Supercapacitor Applications (with Material Size When Cited)	Specific Capacitance [F/g_material_]	Capacitance Retention vs. Cycles	Energy Density [Wh/kg]	Power Density [W/Kg]	Ref.
Ag (5.898 ± 0.108 nm)@rGO||rGO (asymmetric device)	44.17 (at 3 A/g)	81.5% after 500 cycles at 5 A/g	11.09 (at 3 A/g)	2670 (at 3 A/g)	[24]
Ag (400–800 nm)/rGO Fiber (72 μm) (symmetric device)	51.4 F/cm^3^ (at 0.5 A/cm^3^)	No significant reduction after 5000 cycles at 1.6 A/cm^3^	21.47 mWh/cm^3^ (at 0.1 A/cm^3^)	50 (at 0.1 A/cm^3^)	[26]
PANI rGO.Ag (symmetric device)	179.6 (at 1 A/g)	87.18% of the initial capacitance after 5000 cycles at 2 A/g	~26 (at 1 A/g)	~2000 (at 1 A/g)	[30]
rGO/PIn (71 nm)/Al_2_O_3_ (59 nm) nanocomposite||rGO (asymmetric device)	38.46 (at 5 A/g)	83% of its initial capacitance after 5000 cycles at 100 m V/s	10 (at 5 A/g)	3880 (at 5 A/g)	[31]
Graphene-Cu_2_O nanocomposite//Cu foil (asymmetric device)	11.94 (at 10 mV/s)	-	6.63 (at 10 mV/s)	-	[32]
rGO (symmetric device)	83.81 (at 0.5 A)	61.94% of the initial capacitance after 1000 cycles at 100 mV/s	0.042 (at 0.5 A)	341.45 (at 0.5 A)	[29]
rGO/PANI (symmetric device)	159.62 (at 0.5 A)	79.15% of the initial capacitance after 1000 cycles at 100 mV/s	0.31 (at 0.5 A)	1178.78 (at 0.5 A)	[29]
rGO/RuO_2_/PANI (symmetric device)	534.63 (at 0.5 A)	85.14% of the initial capacitance after 1000 cycles at 100 mV/s	1.29 (at 0.5 A)	215.35 (at 0.5 A)	[29]
rGO nanosheets-AuNPs (5–20 nm) @PANI (symmetric device)	212.8 (at 1 A/g)	86.9% of the initial capacitance after 5000 cycles at 2 A/g	7.52 (at 0.5 A/g)	126.5 (at 0.5 A/g)	[28]
Steel-rGO@PANI-DBSA-PLA (symmetric device)	134 (at 1 A/g)	77% of its initial capacitance after 5000 cycles at 1 A/g	10.5 (at 10 A/g)	2.21 (at 10 A/g)	[33]
Ag-rGO@PANI-DBSA-PLA (symmetric device)	133 (at 1 A/g)	91% of its initial capacitance after 5000 cycles at 1 A/g	7 (at 1 A/g)	300 (at 1 A/g)	This work

## Data Availability

The datasets used and/or analyzed during the current study are available from the corresponding author upon reasonable request.

## Supplementary Materials

The following supporting information can be downloaded at: https://www.mdpi.com/article/10.3390/nano14201681/s1. Figure S1: FT-IR spectra of Ag/rGO nanomaterial, and GO.
