# Supplementary material for: Silver-Doped Reduced Graphene Oxide/PANI-DBSA-PLA Composite 3D-Printed Supercapacitors
Source: Nanomaterials (Basel). 2024 Oct 20;14(20):1681. doi: 10.3390/nano14201681 (PMC11514729; doi:10.3390/nano14201681)
Supplement: Supplementary file 1 [file nanomaterials-14-01681-s001.zip › nanomaterials-3237270-supplementary.pdf]

Supplementary Materials

Silver-Doped Reduced Graphene Oxide/PANI-DBSA-PLA Composite 3D-Printed Supercapacitors

Claudia Cirillo [1,2,*], Mariagrazia Iuliano [1,2], Davide Scarpa [1,2], Pierpaolo Iovane [3], Carmela Borriello [3], Sabrina Portofino [3], Sergio Galvagno [3] and Maria Sarno [1,2]

[1] Department of Physics “E.R. Caianiello”, University of Salerno, Via Giovanni Paolo II, 132-84084 Fisciano, Italy; maiuliano@unisa.it (M.I.); dscarpa@unisa.it (D.S.); msarno@unisa.it (M.S.)
[2] NANO_MATES Research Centre, University of Salerno, Via Giovanni Paolo II, 132-84084 Fisciano, Italy
[3] Nanomaterials and Devices Laboratory (SSPT-PROMAS-NANO), ENEA, Italian National Agency for New Technologies, Energy and Sustainable Economic Development, Piazzale E. Fermi 1, 80055 Portici, Italy; pierpaolo.iovane@enea.it (P.I.); carmela.borriello@enea.it (C.B.); sabrina.portofino@enea.it (S.P.); sergio.galvagno@enea.it (S.G.)
* Correspondence: clcirillo@unisa.it; Tel.: +39-089-964335

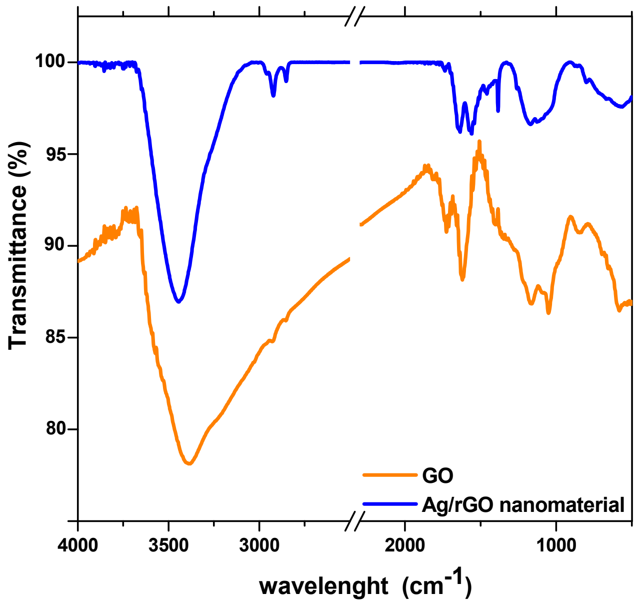

Figure S1. FT-IR spectra of Ag/rGO nanomaterial, and GO.
